# MiR-21 Is under Control of STAT5 but Is Dispensable for Mammary Development and Lactation

**DOI:** 10.1371/journal.pone.0085123

**Published:** 2014-01-30

**Authors:** Yonatan Feuermann, Keunsoo Kang, Avi Shamay, Gertraud W. Robinson, Lothar Hennighausen

**Affiliations:** 1 Laboratory of Genetics and Physiology, National Institute of Diabetes and Digestive and Kidney Diseases, National Institutes of Health, Bethesda, Maryland, United States of America; 2 Animal Science Departments, The Volcani Center, The Ministry of Agriculture, Bet Dagan, Israel; University of Kansas School of Medicine, United States of America

## Abstract

Development of mammary alveolar epithelium during pregnancy is controlled by prolactin, through the transcription factors STAT5A/B that activate specific sets of target genes. Here we asked whether some of STAT5's functions are mediated by microRNAs. The *miR-21* promoter sequence contains a *bona-fide* STAT5 binding site and *miR-21* levels increased in HC11 mammary cells upon prolactin treatment. *In vivo miR-21* was abundantly expressed in mammary epithelium at day 6 of pregnancy. Analysis of mice lacking *miR-21* revealed that their mammary tissue developed normally during pregnancy and dams were able to nurse their pups. Our study demonstrated that although expression of miR-21 is under prolactin control through the transcription factors STAT5A/B its presence is dispensable for mammary development and lactation.

## Introduction

Cytokines control a plethora of biological functions, many of them mediated by the JAK-STAT signal transduction pathway. The transcription factors Signal Transducer and Activator of Transcription 5A and 5B (collectively referred to as STAT5) are key executors of cytokine signaling, including prolactin, growth hormone, erythropoietin, and interleukins [1]. STAT5 controls different facets of mammary development, from the establishment and maintenance of alveolar progenitors during puberty to alveolar differentiation during pregnancy [2]–[4]. The complexity of defects observed in the absence of STAT5 suggests the presence of a network of downstream mediators that effects a variety of physiological functions [5]. MicroRNAs are small non coding RNA molecules, which modulate cell functions via post transcriptional regulation of mRNAs [6]. Like most genes, microRNA genes are regulated by transcription factors and are expressed in tissue and developmental stage specific patterns. For example, the expression of the *miR-21* gene is suppressed by STAT3 in glioma cells [7] but upregulated by STAT3 in multiple myeloma cells [8]. These findings open the possibility that the same microRNA can be differentially controlled by the same transcription factor in different tissues or development stages.

STAT5 is essential for prolactin induced mammary gland development during pregnancy [2], [3]. While multiple STAT5 regulated genes have been discovered [1], only a few microRNAs, among them *miR 17–92* and *miR-15/16* [9], [10] have been linked directly to STAT5. MicroRNA-21 (*miR-21*) is conserved in mammals and it is located within the gene encoding the transmembrane protein 49 (*VPM1*) in human [11] and mouse [8]. MicroRNA profiling of different cancer types revealed that *miR-21* is up regulated in several cancers, including those of breast, prostate and lung [12], [13]. MiR-21 has been shown to target the expression of phosphatase and tensin homologue (*PTEN*) in hepatocellular cancer [14], programmed cell death 4 (*PDCD4*) in colorectal cancer [15] and Sprouty2 (*SPRY2*) in cardiocytes [16]. Notably, transgenic mice expressing active PTEN specifically in mammary epithelium exhibited a decrease in the number of alveolar epithelial cells [17]. Moreover, it has been suggested that low levels of the functional PDCD4 protein may be essential for cell proliferation, and that accumulation of the protein in nuclei may negatively regulate cell proliferation [18], [19]. SPRY2 has been described as a negative regulator of branching morphogenesis [16]. A recent study demonstrated that IL-6 through STAT3 activates the *miR-17/92* cluster [20] and *miR21* [21]. In contrast, expression of the *miR-21* gene is suppressed by STAT3 in glioma cells [7]. Here we asked whether the STAT3 target gene *miR-21* is also recognized and controlled by STAT5.

## Materials and Methods

### Mice

Animals were handled and housed in accordance with the guidelines of NIH and all experiments were approved by the Animal Care and Use Committee of The National Institute of Diabetes and Digestive and Kidney Diseases (NIDDK) (Permit: ASP K089-LGP-11). All efforts were made to minimize suffering. Generation of *miR-21^−/−^* mice was previously described [22]. Mice with a deletion of the *Stat5* locus in mammary epithelial cells have previously been reported by Yamaji et al [2].

### MicroRNA extraction and analysis

miRNA was extracted with miRNeasy system for purifying total RNA (Qiagen). Expression analysis was performed by using TaqMan probes (Applied Biosystems; Foster City, CA) for real-time PCR. cDNA was synthesized from total RNA using gene-specific primers according to the TaqMan MicroRNA Assay protocol (Applied Biosystems; Foster City, CA). Reverse transcriptase reactions contained 10 ng of RNA samples, 50 nM stem-loop RT primer, 1× RT buffer, 0.25 mM each of dNTPs, 3.33 U/µl MultiScribe reverse transcriptase and 0.25 U/µl RNase inhibitor (cDNA Archive kit from Applied Biosystems). The 7.5 µl reactions were incubated for 30 min at 16°C, 30 min at 42°C, 5 min at 85°C and then held at 4°C. Real-time PCR was performed using an Applied Biosystems 7300 Sequence Detection system. The 20 µl PCR included 0.67 µl RT product, 1× TaqMan Universal PCR master mix and 1 µl of primers and probe mix of the TaqMan MicroRNA Assay protocol (Applied Biosystems; Foster City, CA). The reactions were incubated in a 96-well optical plate at 95°C for 10 min, followed by 40 cycles of 95°C for 15 sec and 60° for 10 min. The CT values were determined using default threshold settings. The threshold cycle (CT) is defined as the fractional cycle number at which the fluorescence passes the fixed threshold.

### RNA extraction and analysis

Total RNA was extracted with the miRNeasy system (Qiagen). Expression analysis was performed using TaqMan probes (Applied Biosystems; Foster City, CA) for real-time PCR. Real-time PCR was carried out on an ABI Prism 7900HT (Applied Biosystems). Individual PCRs were performed in triplicate on samples using the housekeeping gene mouse beta-actin for normalization and experimental probes to obtain average threshold cycle (CT) values. The threshold cycle is defined as the fractional cycle number at which the fluorescence passes the fixed threshold. Average beta-actin CT values were subtracted from experimental CT values to obtain delta CT values. Delta CT values were then calculated by subtracting experimental sample delta CT values from the control sample delta CT. Relative gene expression was calculated using 2 delta CT.

### HC-11 cell culture

Mouse mammary HC-11 epithelial cells were cultured in DMEM/F-12 medium supplemented with 5% FBS, 5 µg/ml insulin, 100 ng/ml hydrocortisone, 100 U/ml penicillin and streptomycin. Cells were plated on 100-mm dishes at a density of 1.0×10^6^ cells and grown at 37°C for approximately 24 h to reach 60–70% confluence. Cells were then cultured for 24 h in DMEM/F-12 medium supplemented with 5 µg/ml insulin and 100 U/ml penicillin. After 24 h prolactin (0.5 µ µg/ml; source?) was added to the cultured medium. After 0.5, 1, 2 and 4 h the cells were harvested and total RNA was extracted.

### RNA-Seq analysis

RNA was prepared for sequencing using the TruSeq RNA Sample Prep Kit, Set A (Illumina catalog # FC-122-1001) according to manufacturer's recommendations. Each sample was prepared from 1 µg total RNA. cDNA was synthesized using random hexamer primers and SuperScript II (Invitrogen). The resulting cDNA libraries were quantified using the QuantiFluor™-ST system and checked for quality and size using the Agilent 2100 Bioanalyzer (High Sensitivity DNA kit, Agilent catalog # 5067-4626). A portion of each library was diluted to 10 nM and stored at −20°C. 2 µl of the 10 nM libraries were diluted and denatured, according to instructions from Illumina. The samples were loaded onto the cBot for clustering on a flow cell according to manufacturer's instructions (Illumina Inc., San Diego, CA) and sequenced using HiSeq 2000 (Illumina). The single-end sequenced tags from biological replicates of both wild-type and *miR-21* ^−/−^ samples were aligned to the mouse reference genome (mm9 assembly) using the TopHat program [23]. Transcript abundances were estimated by means of FPKM (fragment per kilobase of exon per million fragments mapped) and differentially expressed genes (DEGs) were initially identified by using the Cufflinks program [24]. To remove potential false-positives, we only regarded the identified genes showing more than 5 FPKM in either *miR-21^+/+^* or *miR-21^−/−^* samples as reliable DEGs. All statistical analyses were performed using a two-tailed unpaired Student's t test. A p-value of 0.05 or less was considered significant. The RNA-seq data are deposited in GEO under accession number (GSE50068).

### Transplantation of mammary epithelia

Transplantations were performed as previously described [4]. In brief, athymic nude mice (3-wk-old) were anesthetized and the proximal part of the inguinal gland containing the mammary epithelium was excised. Small pieces of mammary tissue from mature virgin female *miR-21^+/+^* or *miR-21^−/−^* mice were transplanted into the cleared fat pads. To assess the complete removal of host epithelia, the excised endogenous glands were processed for whole mount staining. Six weeks after transplantation, fat pads were harvested from virgin hosts and processed for whole mount staining in carmine alum.

### Chromatin immunoprecipitation assay

Chromatin immunoprecipitation assay was performed as previously described [25], with some modifications. Enriched primary mammary cells were cultured in complete EpiCult-B medium (supplemented with 5% fetal bovine serum). Once the cells had reached 80% confluency they were starved for 5 h, immediately after starvation they were stimulated with prolactin for 1 h. Cells were cross-linked using 1% formaldehyde (Protocol Formalin; Fisher Scientific). Cell lysates were sonicated and immunoprecipitated with α-STAT5 antibody (R&D Systems, Minneapolis, MN) or rabbit serum as control (Upstate Biotechnology). Immunoprecipitated DNA was eluted, amplified by real-time PCR using a 7900 HT fast real-time PCR system (Applied Biosystems) and analyzed using SDS2.3 Software (Applied Biosystems). Sequence-specific primers used for the proximal predicted GAS sites in the *miR-21* promoter as well as *Socs2* and *Igf1* GAS sites were previously described [25]. *Socs2*: forward primer 5′-GGAGGGCGGAGTCGCAGGC-3′, reverse primer 5′-GACTTGGCAAGAGTTAACCGTC-3′, *Socs2*: forward 5′-GCATATGTCTCTGAAAGGGGTGA-3′ and reverse 5′-GGCACAAGCTAGCCGATGGTTAG-3′; for the *miR-21* GAS sequence, forward primer 5′- TGAGAAGTCCCACATTTATCACC-3′, reverse primer 5′-GAGAGGGAGGGCAGTTTCTT-3′. Primers for a region outside of a suspected STAT5 binding region were forward 5′-AGAAGGGGCGAGTTCTTAGC-3′, reverse 5′-CTGATCTCCTGCCTCTACCG-3′.

### Locked nucleic acid (LNA)-modified-in situ hybridization for miR-21

Locked nucleic acid (LNA)-modified, digoxigenin (DIG)-labeled probes complementary to mouse mature miRNAs were obtained from Exiqon, Inc (Woburn, MA). Sections of *Stat5a/b^fl/fl^* mammary tissue from 6 days pregnant mice were deparaffinized and treated with proteinase K for 20 min (10 µg/ml). After washing in PBS, sections were re-fixed in 4% paraformaldehyde for 10 min, washed twice in PBS, and prehybridized for 1 h in hybridization buffer (Roche, Mannheim, Germany). Tissues were hybridized overnight in the presence of 0.4 pmol/µl probe at 50°C. Slides were washed twice in 2× SSC at 37°C and finally washed in a high stringency 50% formamide-2× SSC at the hybridization temperature. Immunological detection was carried out using anti-DIG Fab conjugated to alkaline phosphatase (Roche, Mannheim, Germany) according to the manufacturer's protocol.

### Statistics

All statistical analyses were performed using a two-tailed unpaired Student's t test. A p-value of 0.05 or less was considered significant.

## Results

### *Mir-21* expression in HC-11 cells after prolactin stimulation

**Figure 1:**
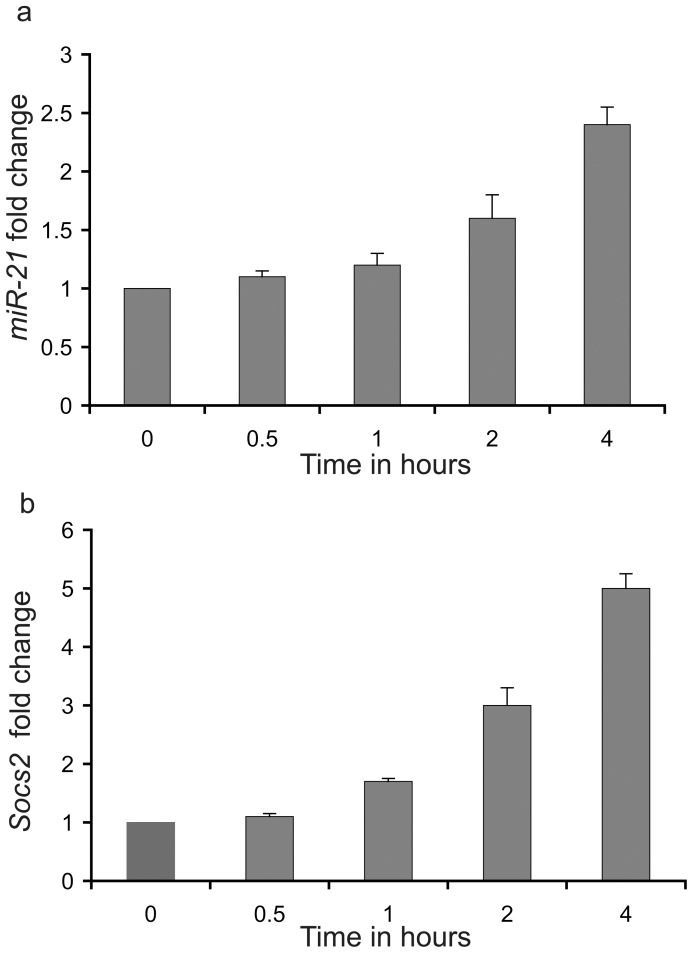
MicroRNA 21 detection in HC-11 cells. (a) Expression of *miR-21* in HC-11 cells at different times of prolactin stimulation (0.5 h, 1 h, 2 h and 4 h). (b) Expression of *Socs2* in HC-11 cells at different times of prolactin stimulation (0.5 h, 1 h, 2 h and 4 h). In each analysis the unstimulated cells were used as the control sample (fold change = 1). The miRNA levels were normalized to U6 levels, mRNA levels were normalized to beta-actin mRNA levels. Values are means ± S.D. for three independent experiments.

HC-11 cells were exposed to prolactin (0.5 µg/ml) for 0.5, 1, 2 and 4 hours. *MiR-21* (Figure 1a) and *Socs2* (Figure 1b) RNA levels were analyzed by real time PCR. mRNA levels for *Socs2*, a known STAT5 target gene, was elevated ∼1.8 and 5 fold after 1 and 4 hours, respectively. *MiR-21* levels increased ∼1.7 fold and 2.5-fold after 2 and 4 hours, respectively.

### *Mir-21* expression in mammary epithelium

**Figure 2:**
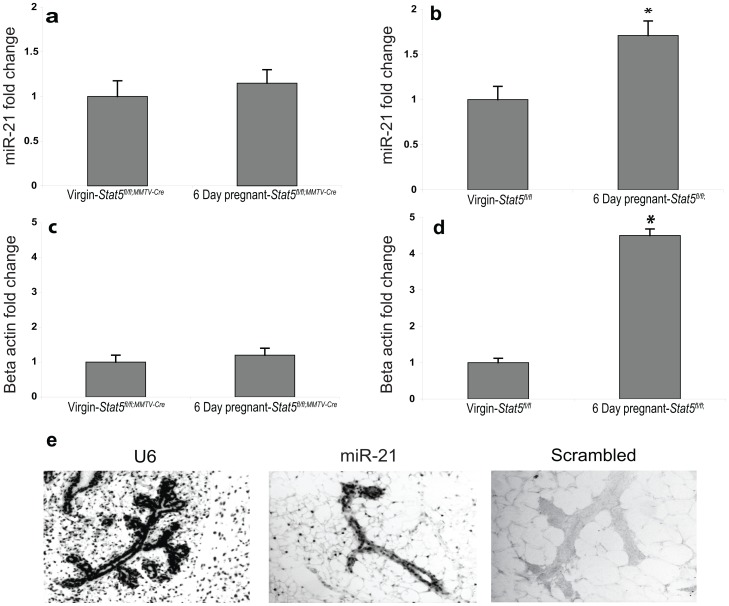
MicroRNA 21 detection in the mammary gland. (a) Expression of *miR-21* in mammary glands from virgin and 6 day pregnant *Stat5^fl/fl^* and *Stat5^fl/fl;MMTV-Cre^* mice. (b) Expression of *miR-21* in *Stat5^fl/fl^* mice in mammary glands from virgin and 6 day pregnant mice. (c) Expression of beta-casein in *Stat5^fl/fl;MMTV-Cre^* mice in mammary glands from virgin and 6 day pregnant mice. (d) Expression of beta-casein in *Stat5^fl/fl^* mice in mammary glands from virgin and 6 day pregnant mice. The miRNA levels were normalized to U6 levels, mRNA levels were normalized to beta-actin mRNA levels. In each analysis the virgin mammary gland was used as the control sample (fold change = 1). Values are means ± S.D. for three independent experiments. (e) Detection of *mir-21* expression in the mammary gland of 6 day pregnant mice. In situ hybridization analysis using DIG-conjugated, LNA-modified DNA probes complementary to U6 (right), miR-21 (middle) and scrambled (left). Detection was performed on formalin-fixed, paraffin-embedded section from 6 day pregnant mammary glands.

MiR-21 promotes cell transformation [26] and proliferation [27], [28]. We chose to analyze miR-21 levels in mammary tissue from day 6 of pregnancy as this stage is characterized by extensive cell proliferation [29]. Compared to virgin tissue, miR-21 levels in control mammary tissue at pregnancy day 6 had increased ∼1.7 fold (Figure 2b). In contrast, miR-21 levels did not change in tissue from *Stat5^fl/fl;MMTV-Cre^* mice, in which STAT5 was deleted in the entire mammary epithelial compartment (Figure 2a). Increased expression of beta-casein (*Csn2*) served as a control for proper STAT5 activation. While *Csn2* mRNA levels where elevated ∼4.5 fold in tissue from pregnant *Stat5^fl/fl^* mice (Figure 1d) they did not change in tissue from *Stat5^fl/flMMTV-Cre^* mice (Figure 2c). In order to validate *miR-21* is expression we used in situ hybridization with locked nucleic acid (LNA) nucleotides, which detects mature miRNAs in tissue sections. Mammary tissues from day 6 pregnant *Stat5^fl/fl^* mice were used to perform the hybridization with miR-21, U6 and scrambled double digoxigenin labeled LAN oligonucleotides. The results demonstrated that *mir-21* is preferentially expressed in the epithelial compartment (Figure 2e).

### STAT5a binds to the *miR-21* gene promoter

**Figure 3:**
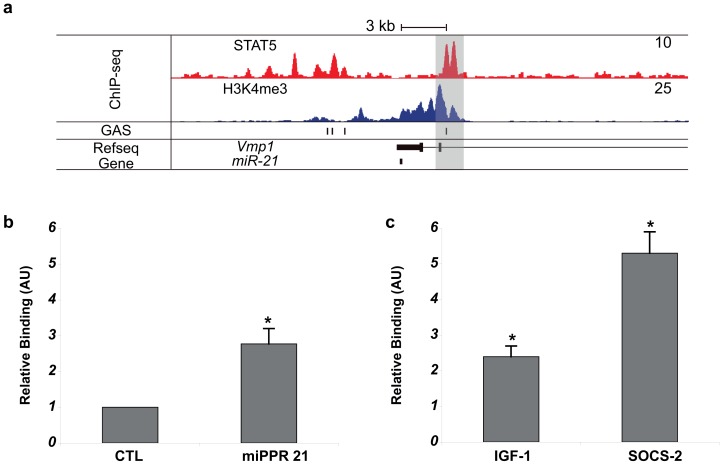
STAT5 binding to the *miR-21* gene promoter. (a) Reanalysis of previous genome-wide STAT5A and H3K4me3 ChIP-seq datasets (GSE40930) performed in mammary gland at parturition suggested that the expression of *miR-21* might be controlled by STAT5 [30]. The first track shows normalized tag density of STAT5 and H3K4me3 ChIP-seq datasets. The second and third tracks represent positions of GAS motifs (TTCnnnGAA) and genes, respectively. *MiR-21* is located within the 3′-UTR of the *Vmp1* gene. Grey bar indicates the STAT5 binding site coincided with a GAS motif. (b) Chromatin immunoprecipitation (ChIP) analysis of binding of STAT5A to the putative GAS sites within the *miPPR21* promoter sequence. (c) ChIP analysis of binding of STAT5A to the GAS sites within the promoter region of *Igf1* and *Socs2* gene. Values are means ± S.D. for three independent experiments.

The *miR-21* gene promoter is located in an intron of the *TMEM49* gene and contains two *bona fide* STAT3 binding sites at approximately 800 bp upstream of the transcriptional start site [8], [11]. Since STAT3 and STAT5 recognize the same GAS motif (TTCnnnGAA) this site likely is also recognized by STAT5. Furthermore, reanalysis of previous genome-wide STAT5A and H3K4me3 ChIP-seq datasets (GSE40930) performed in mammary gland at parturition, clearly showed that STAT5A binds to the *miR-21* promoter and aligns with the presence of H3K4me3 [30] (Figure 3a). In order to confirm the STAT5A binding to the *miR-21* gene promoter in mammary tissue, primary mammary epithelial cells were stimulated with prolactin for 1 h followed by ChIP analysis. Real-time PCR analysis of DNA extracted after immunoprecipitation with anti-STAT5A antibodies, confirmed a 2.5 fold increased binding of STAT5A to the GAS motif in the *miPPR21* compared to the untreated sample (Figure 3b). Binding to GAS sites in the promoter of the *Igf1* and *Socs2* genes, which both are STAT5 target genes, was enhanced by ∼2.5 fold and ∼5 fold, respectively (Figure 3c).

### Assessing the role of miR-21 during mammary gland development

**Figure 4:**
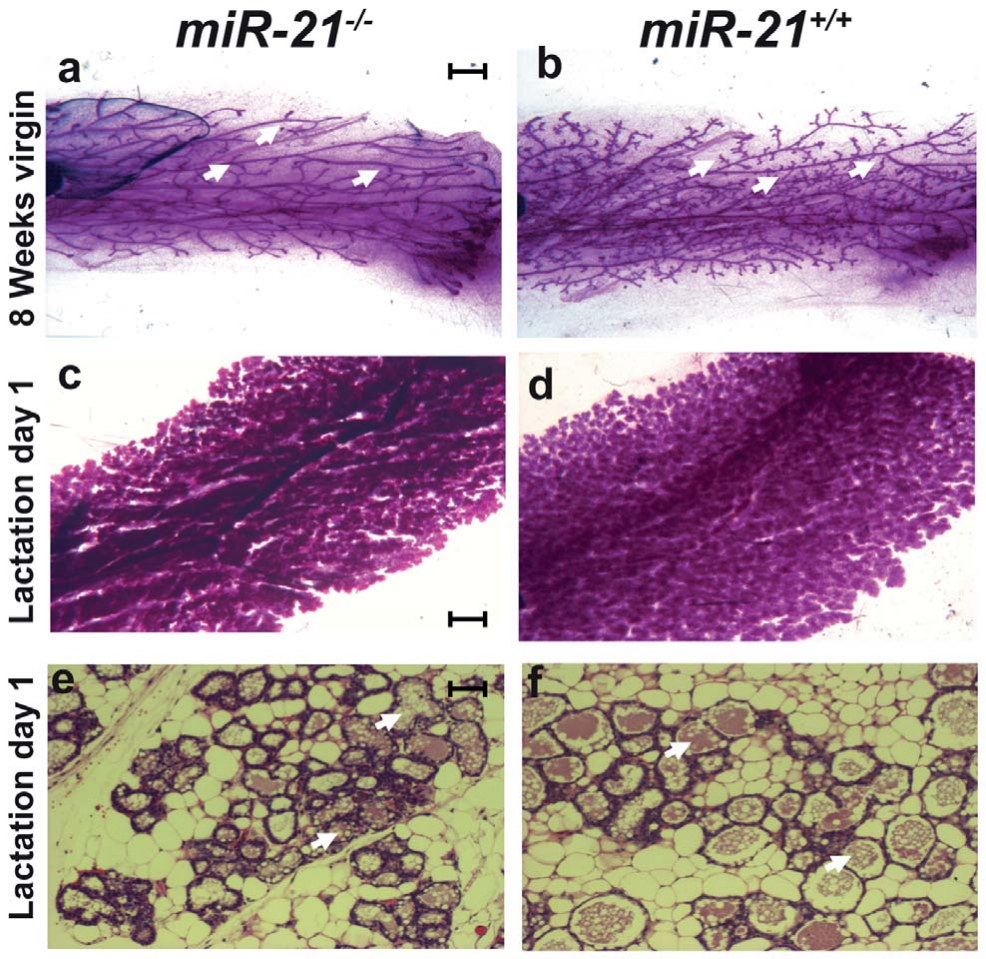
Mammary gland whole mounts and H&E analysis. Mammary glands from 8 weeks old virgin mice revealed fewer secondary ductal branching (marked with white arrows) in *miR-21^−/−^* (a) compared to *miR-21^+/+^* mice (b). No differences were detected in mammary glands harvested at the first day of lactation from *miR-21^−/−^* (c) and *miR-21^+/+^* dams (d). No differences were detected in the H&E staining in mammary gland harvested at the first day of lactation from *miR-21^−/−^* (e) and *miR-21^+/+^* animals (f). White arrows indicate lumina. Scale bar indicates 2 mm in a, b, c and d, 100 µm in e and f.

**Figure 5:**
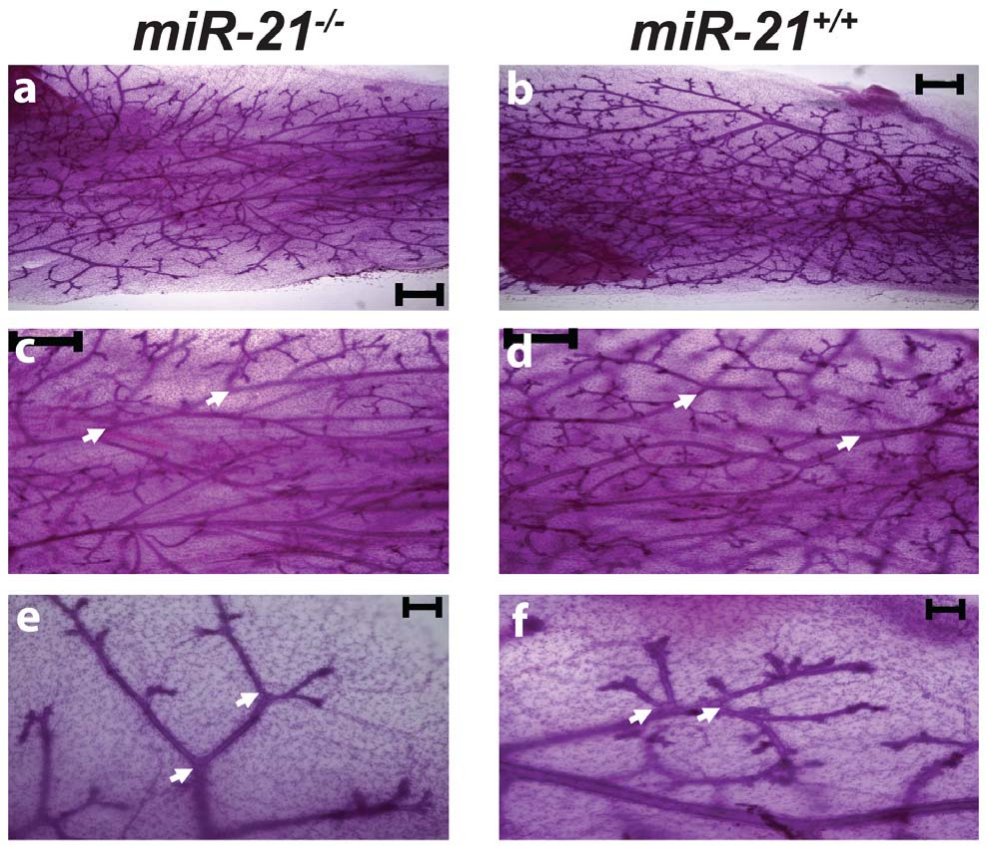
Whole mount analyses of transplanted mammary tissue from *miR-21^−/−^* and *miR-21^+/+^* mice. Whole mount analyses of transplanted mammary tissue of *miR-21^−/−^* (panels a, c and e) and *miR-21^+/+^* (panels b, d and f) virgin donors harvested 6 weeks after transplantation. Higher magnification of the whole mounts shown in middle and lower panels. White arrows indicate secondary branching points. Scale bar indicates 500 µm in a, b, c and d, 100 µm in e and f.

Next we investigated mammary development in *miR-21^−/−^* mice from puberty to lactation. M*iR-21^−/−^* females were able to nurse their pups until weaning. Mammary whole mount analyses from 8 week-old virgins and dams at their first day of lactation confirmed normal mammary alveolar structures (Figure 4 a–d). Histological analysis of *miR-21^−/−^* mammary epithelium at parturition displayed all features of secretory epithelium (Figure 4 e–f). Notably, fewer secondary ductal branches were seen in 8 week-old *miR-21^−/−^* mice (Figure 4 a–b). In order to examine epithelial cell autonomy, *miR-21^−/−^* mammary epithelium was transplanted into control mice. When harvested 6 weeks after transplantation, the *miR-21^−/−^* transplants did not exhibit any defects in the secondary branching pattern suggesting that systemic defects are responsible for this delay (Figure 5).

### Target analysis of miR-21 in the mammary gland

**Figure 6:**
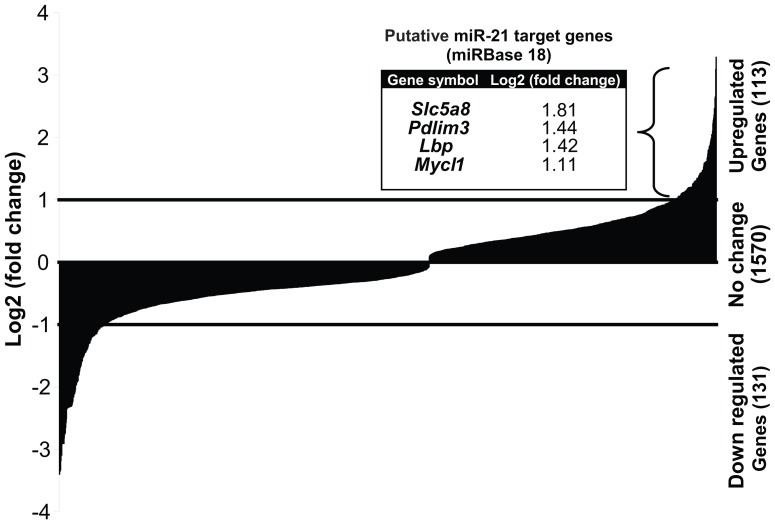
Analysis of differentially expressed genes in the mammary gland of *miR-21^−/−^*. RNA-Sequencing analysis resulted in a total of 1814 differentially expressed which were detected. Out of the statistically significant differentially expressed genes in *miR-21^−/−^* over the *miR-21^+/+^* a total of 113 genes were up regulated and 131 were down regulated in *miR-21^−/−^* with at least 2 fold changes over the *miR-21^+/+^*. Out of the up regulated genes, 4 genes are considered to be *miR-21* putative targets (the gene names and fold changes are indicated in the insert).

To identify miR-21 target genes we analyzed the transcriptome of mammary tissue harvested from *miR-21^−/−^* and *miR-21^+/+^* mice at 8 weeks of age. Overall, 1814 differentially expressed annotated genes were detected. Statistically significant differentially expressed genes in *miR-21^−/−^* over *miR-21^+/+^* with fold changes greater than 2 were considered. This analysis resulted in 113 up regulated genes and 131 down regulated genes in *miR-21^−/−^* (Figure 6, Table S1, GEO accession number-GSE50068). The 113 up regulated genes were aligned with the mmu-miR-21 putative targets from microRNA.org [35]. Only conserved target genes with a mirSVR score lower than −0.1 were considered [31] . This analysis resulted in 4 novel miR-21 putative target genes; solute carrier family 5 (iodide transporter) member 8 (*Slc5a8*), lipopolysaccharide binding protein (*Lbp*), V-myc myelocytomatosis viral oncogene homolog 1, lung carcinoma derived (*Mycl1*), PDZ and LIM domain 3 (*Pdlim3*) (Table S2). Analysis of the differential expression levels of validated *miR-21* targets *sprouty homolog 2* (*Spry2*) [16], programmed cell death 4 (*Pdcd4*) [26] and phosphatase and tensin homolog (*Pten*) [14] did not show any changes in their expression levels (Table 1).

**Table 1:** MiR-21 Target Analysis in virgin mammary gland.

Gene	* *miR-21^+/+^*	* *miR-21^−/−^*	Log2 (Fold change)	P-value
*Pdcd4*	43.9	45.6	0.056	0.46
*Pten*	31.1	26.3	−0.24	0.03
*Spry2*	8.3	9.8	0.24	0.22

* FPKM (normalized expression level).

## Discussion

Development of mammary tissue during pregnancy is fully dependent on prolactin and its downstream transcriptional executor STAT5, which activates a differentiation program culminating in lactation [3], [32]. Here we identify *miR-21* as a STAT5 target gene both in a mammary epithelial cell line (HC-11) and in the developing mammary gland. We also show STAT5 binding to the *miR-21* promoter (*miPPR-21*) in primary mammary cells. The ability of STAT5 to bind to the *miPPR-21* gene and activate its transcription was also reported for Jurkat cells, an immortalized line of T lymphocytes [33]. Based on our study and other reports [8], it is likely that the *miPPR-21* gene is recognized by either STAT3 or STAT5 and thus can be activated by several distinct cytokines, which even might initiate opposing biological programs. For example, prolactin activates STAT5 to control proliferation, differentiation and survival [2], while STAT3 is activated by IL-6 and LIF to control cell death during involution [34]. If STAT3 as well as STAT5 activate *miR-21* gene expression during diametrically opposed developmental programs (survival versus death) it can be predicted that *miR-21* serves as a common STAT cytokine-induced microRNA that targets different genes at different developmental stages during mammary gland development. Although the *miR-21* gene is under STAT5 control, pregnancy mediated mammary development of *miR-21* deficient mice (*miR-21^−/−^*) was normal and these mice delivered and lactated normally and no abnormalities were detected in pup growth.

While attenuated secondary mammary ductal branching was observed in *miR-21* ^−/−^ mice during puberty, *miR-21^−/−^* epithelium transplanted into fat pads of wild type mice developed normally. It is therefore likely that either suboptimal ovarian function or impaired signaling from the stroma is responsible for impaired ductal branching. Analysis of RNA-seq data which was performed on 8–10 week-old *miR-21^−/−^* and *miR-21^+/+^* mice did not exhibit any differences in well-known *miR-21* target gens such as *Spry2*, *Pdcd4* and *Pten* (Table 1, *GSE50068*). Furthermore, the distribution of mammary epithelial cell lineages (luminal, myoepithelial and stem cells) did not show any difference between the *miR-21* ^−/−^ mammary and control tissue (data not shown).

In summary, STAT5 controls different aspects of mammary development, from establishment and maintenance of alveolar progenitors during puberty to alveolar differentiation during pregnancy. While some of the developmental steps are controlled by STAT5-dependent protein-encoding genes, others might be under the influence of microRNAs. However, *miR-21*, which is regulated by STAT5, is not involved in the regulation of the mammary gland development or lactation.

## Supporting Information

Table S1 **RNA-Seq summery of statistically significant differentially expressed genes in miR-21^−/−^ over miR-21^+/+^ with fold changes greater than 2.**
(XLS)

Table S2 **miR-21 target analysis.**
(XLS)
